# Statin-Associated Myopathy: Emphasis on Mechanisms and Targeted Therapy

**DOI:** 10.3390/ijms222111687

**Published:** 2021-10-28

**Authors:** Pierandrea Vinci, Emiliano Panizon, Letizia Maria Tosoni, Carla Cerrato, Federica Pellicori, Filippo Mearelli, Chiara Biasinutto, Nicola Fiotti, Filippo Giorgio Di Girolamo, Gianni Biolo

**Affiliations:** 1 Clinica Medica, Cattinara Hospital, Department of Medical Surgical ad Health Science, University of Trieste, 34149 Trieste, Italy; emiliano.panizon@asugi.sanita.fvg.it (E.P.); letizia.tosoni@gmail.com (L.M.T.); arancioca@hotmail.it (C.C.); Federica.pellicori@live.it (F.P.); filippome@libero.it (F.M.); fiotti@units.it (N.F.); fgdigirolamo@units.it (F.G.D.G.); biolo@units.it (G.B.); 2 SC Assistenza Farmaceutica, Cattinara Hospital, Azienda Sanitaria Universitaria Integrata di Trieste, 34149 Trieste, Italy; chiara.biasinutto@gmail.com

**Keywords:** statin myopathy, SAMS mechanisms, SAMS management

## Abstract

Hyperlipidemia is a major risk factor for cardiovascular morbidity and mortality. Statins are the first-choice therapy for dyslipidemias and are considered the cornerstone of atherosclerotic cardiovascular disease (ASCVD) in both primary and secondary prevention. Despite the statin-therapy-mediated positive effects on cardiovascular events, patient compliance is often poor. Statin-associated muscle symptoms (SAMS) are the most common side effect associated with treatment discontinuation. SAMS, which range from mild-to-moderate muscle pain, weakness, or fatigue to potentially life-threatening rhabdomyolysis, are reported by 10% to 25% of patients receiving statin therapy. There are many risk factors associated with patient features and hypolipidemic agents that seem to increase the risk of developing SAMS. Due to the lack of a “gold standard”, the diagnostic test for SAMS is based on a clinical criteria score, which is independent of creatine kinase (CK) elevation. Mechanisms that underlie the pathogenesis of SAMS remain almost unclear, though a high number of risk factors may increase the probability of myotoxicity induced by statin therapy. Some of these, related to pharmacokinetic properties of statins and to concomitant therapies or patient characteristics, may affect statin bioavailability and increase vulnerability to high-dose statins.

## 1. Introduction

Hyperlipidemia is a major risk factor for cardiovascular morbidity and mortality. Statins [3-hydroxy-3-methylglutaryl coenzyme A reductase (HMG-CoA) inhibitors] are the first-choice therapy for dyslipidemias and are considered the cornerstone of Atherosclerotic cardiovascular disease (ASCVD) prevention [1]. Despite the benefits on cardiovascular outcomes, the compliance to statin therapy is often inadequate, with non-adherence to treatment reaching up to 50% of patients [2,3]. 

A meta-analysis of 15 statin studies observed a 45% increase in all-cause mortality and a 15% increase in Cardiovascular diseases (CVDs) in patients taking <80% of their prescribed statin therapy when compared to compliant patients [4]. High discontinuation rates, mainly due to the development of drug-related side effects, contributed to this phenomenon [2,5]. Statin-associated muscle symptoms (SAMS) are the most common statin side effects associated with treatment discontinuation. 

Many hypotheses have been formulated in order to give an explanation about SAMS, ranging from low cholesterol levels in muscle cell membranes to reduction of intermediate products of the mevalonate pathway, such as prenylated proteins, dolichols, and electron transport chain proteins. Dolichols promote protein N-glycosylation, ensuring expression of receptors and the production of structural proteins in muscle cells [6,7]. Farnesyl pyrophosphate and geranylgeranyl pyrophosphate, other intermediates of the mevalonate pathway, are essential for the prenylation of lamins and guanosine-5’-triphosphate (GTP)-binding proteins. These proteins, in fact, need prenylation in order to control cytoskeleton organization and intracellular vesicular transport, promoting muscles’ cell function, growth, and differentiation [6,7,8,9]. The decline in protein prenylation leads also to elevated cytoplasmic Ca^2+^, which seems correlated to damages of ryanodine receptors (RYRs). Calcium entry also may trigger activity of proteases, such as caspases and calpains [10,11,12,13]. In view of this, statins are no longer capable of inducing apoptosis in muscle cells once the protein geranylgeranylation is restored by mevalonate, farnesol, or geranylgeraniol [14].

The purpose of this manuscript is to give an update on SAMS diagnosis and pathophysiological mechanisms, focusing on the most effective strategies for statin intolerance clinical management, including new emerging alternative therapies. This review aims to improve diagnostic and therapeutic SAMS approaches in order to increase statin adherence and reduce cardiovascular events.

## 2. Definition

Statin-associated muscle symptoms range in severity from mild-to-moderate muscle pain, weakness, or fatigue to potentially life-threatening rhabdomyolysis. Rhabdomyolysis is the rarest and most serious muscle-related adverse event associated with statin treatment and is defined as muscle pain accompanied by Creatine kinase (CK) levels > 10× upper limit of normal (ULN) and evidence of renal function impairment or CK levels > 50× ULN [15]. The most common SAMS are myalgia; weakness; lower back, proximal muscle and tendon pain; and nocturnal muscle cramping involving large muscle groups. Symptoms and/or biochemical abnormalities frequently appear early after beginning treatment, disappear after discontinuation, and recur within days to weeks after rechallenge of statin [16,17,18]. Diagnosis of SAMS may be difficult, lacking a definitive diagnostic test. CK, a biomarker of muscle damage, is commonly used to detect skeletal muscle damage and its severity. However, CK levels are frequently normal in symptomatic patients assuming statins, while may be increased in asymptomatic patients [19,20]. For these reasons, CK is a non-sensitive biomarker for statin-induced myopathy, but is currently used in the evaluation of SAMS due to the absence of other specific laboratory tests. Among the different SAMS classifications, the European Atherosclerosis Society (EAS) clinical assessment algorithms include the nature of the muscle symptoms, increase in CK levels, and temporal association with beginning of therapy, suspension, and rechallenge. All kinds of muscle pain were considered as “muscular symptoms”, classified according to CK values and divided among the presence or absence of high levels of this biomarker of muscle damage [21]. The American College of Cardiology (ACC), the American Heart Association (AHA) [22], and the National Lipid Association (NLA) focused mainly on symptoms and on CK increase, and less on clinical diagnostic score, as was proposed by the Canadian Working Group (CWG) [23]. Despite all these classifications proposing CK levels in the spectrum of SAMS disease, symptoms frequently occur without biomarker elevation. Therefore, these definitions are useful for quantifying SAMS in clinical trials rather than in clinical practice, where diagnosis depends on subjective symptoms.

## 3. Prevalence

SAMS, ranging from mild-to-moderate muscle pain, weakness, or fatigue to potentially life-threatening rhabdomyolysis, are reported by 10% to 25% of patients receiving statin therapy [16,24,25,26]. The real incidence is still controversial; in fact, randomized clinical trials reported a 1.5–5% incidence of statin-induced myopathy, while in the clinical practice the frequency appears to be higher [27,28]. In the PROSISA study, which addressed the prevalence of reported SAMS by the lipid specialists in 23 selected Italian Lipid Clinics, SAMS were reported by 9.6% of the cohort. This percentage was higher amongst women (10.7% vs. 8.7% in men, *p* < 0.0001), younger subjects (10.5% vs. 8.2% in subjects ≥ 65 years, *p* < 0.0001), and subjects reporting physical activity (11.5% vs. 9.8% in inactive subjects, *p* = 0.0127) [29]. In a six-month double-blind randomized control study conducted in 420 healthy volunteers, 9.4% of subjects taking atorvastatin 80 mg daily therapy experienced myalgia compared to 4.6% of placebo patients [30]. In the Anglo-Scandinavian Cardiac Outcomes Trial-Lipid Lowering Arm (ASCOT-LLA), a double-blind randomized control trial, the frequency of muscle-related adverse events did not differ between patients on atorvastatin 10 mg daily (1.26%) compared to placebo (1%) [31]. Similarly, the Justification for the Use of Statins in Prevention: an Intervention Trial Evaluating Rosuvastatin (JUPITER) trial, showed no significant differences in the rates of adverse events between participants allocated either to rosuvastatin (20 mg) or to placebo, except for a slightly higher rate of muscle symptoms with rosuvastatin as myalgia (4.0%, rosuvastatin vs. 3.0%, placebo), muscular weakness (0.5%, rosuvastatin vs. 0.3%, placebo), and myopathy (0.04% rosuvastatin vs. 0.05% placebo) [32]. On the other hand, in observational studies, SAMS incidence was higher, affecting up to 10–33% of statin users, [24,25] representing the main cause of discontinuation therapy. In the Understanding Statin Use in America and Gaps in Patient Education (USAGE) study, a cross-sectional internet-based survey, 60% of the cohort reported SAMS [33].

## 4. Diagnosis

The clinical approach to patients with possible SAMS can be summarized in three phases. The first one is patient assessment, which consists of a general evaluation of clinical features, such as statin type and dosage, muscular symptoms in terms of location, timing of onset, and biomarkers levels. The second phase is dechallenge, which consists of the discontinuation of therapy, generally for 2–4 weeks, in order to verify the eventual remission of symptoms. The last phase is rechallenge, either with the same statin or another. Recurring muscular symptoms after rechallenge suggest the causal association between statin and SAMS. The absence of a univocal definition of statin intolerance and the lack of a clear classification and a validated diagnostic test for the condition make it difficult to diagnose SAMS. Many questionnaires have been proposed to help clinicians in identifying and managing this condition. The Statin Muscle Safety Task Force of NLA proposed the SAMS Clinical Index (SAMS-CI), which scores SAMS as probable (score 9–11), possible (score 7–8), and unlikely (score 2–6) based on location, pattern, timing of onset, and timing of improvement of symptoms after statin withdrawal [34,35]. In order to assess patient experience during therapy and to promote patient–physician communication the Statin Experience Assessment Questionnaire (SEAQ) has also been proposed. SEAQ is a self-evaluation questionnaire composed of seven questions regarding muscular symptoms, in which patients provide a numeric answer through a scale from 0 to 10, and two questions about the eventual discontinuation of therapy [36]. Another available questionnaire is the Patient and Provider Assessment of Lipid Management (PALM). This survey evaluates statin experience through medical prescription, patient reasons for declining or discontinuing statins, and beliefs about statins and cardiovascular disease risk [37].

### Risk Factors

Many risk factors, both in terms of patient and treatment characteristics, seem to increase risk of developing SAMS. Focusing on the patient side, female gender, old age, lower body mass index, Asian ethnicity, hypothyroidism, low vitamin D levels, diabetes mellitus, renal, hepatic and muscle diseases, carnitine palmitoyl transferase II deficiency, and frailty appear to increase the likelihood of SAMS onset. The pool of risk factors also includes exogenous factors such as alcohol consumption, heavy exercise and major surgery [38,39,40,41], and genetic markers that may increase the risk of statin toxicity [26]. Genetic variants of Cytochrome P (CYP) isoenzymes might affect the individual response to statin therapy. Polymorphisms in the Solute carrier organic anion transporter, SLCO1B1, appear to be one of the principal genetic factors for risk for developing SAMS [42,43]. SLCO1B1 has been shown to play a relevant role in simvastatin-induced risk of myopathy after increasing the statin’s plasma level, without similarly affecting tolerance to atorvastatin and rosuvastatin [44,45].

Literature has extensively analyzed the correlation between SAMS and statin therapy. The Prediction of Muscular Risk in Observational Conditions (PRIMO) study was an observational survey of muscular symptoms onset in an unselected hyperlipidemic population treated with high-dose statins (atorvastatin, fluvastatin, and simvastatin). Fluvastatin was associated with the lowest rate of muscular symptoms (5.1% of patients), in contrast with a high dosage simvastatin (18.2%) [46]. The Study of Effectiveness of Additional Reduction in Cholesterol and Homocysteine (SEARCH) reported a lower risk of statin-related myopathy with 20 mg of simvastatin compared to 80 mg of simvastatin [47].

The Effect of Statins on Skeletal Muscle Function and Performance (STOMP) study evaluated the effect of high-dose atorvastatin (80 mg) on muscle strength during 6 months of follow up without finding any measurable change in muscle function except for mild increases in plasma CK levels. Other studies compared statin posology and plasma concentration to the incidence of myopathy [48,49]. Despite the dose-dependent nature of SAMS having been demonstrated by several studies as well as in clinical practice [50,51,52] the Treating to New Targets (TNT) study, which included 10,001 patients, found no difference in the rate of statin myalgia between high-dose and low-dose atorvastatin treatment regimens [53]. Similar results have been pointed out by Naci et al. [54]. It seems more likely that high-dose statin therapy has a synergistic effect with other risk factors of SAMS. Thus, statin dose alone may not be a major contributor to the development of muscular symptoms.

The role of lipophilicity is also controversial. It is a common belief that lipophilic statins (simvastatin, lovastatin, atorvastatin, fluvastatin) can increase myotoxicity because of their ability to diffuse in a non-selective way across the cell membranes of extra-hepatic tissues compared to the hydrophilic ones (rosuvastatin, pravastatin) [55,56,57,58]. A meta-analysis by J.C. Irwin et al. found, with the exception of the Goal Achievement after Utilizing an anti-PCSK9 antibody in Statin Intolerant Subjects-3 (GAUSS-3) study, no significant association between either lipophilic or hydrophilic statins and the risk of skeletal muscle events. Another meta-analysis of statin randomized controlled trials (RCTs) also found limited evidence that lipophilic statins were associated with a greater risk of SAMS [55]. A possible explanation of the observed increase in risk of SAMS with lipophilic statins treatment in clinical practice may be due to the high drug–drug interactions that they are involved into compared to hydrophilic statins [39]. The principal reason of increasing plasma statin concentration is represented by co-prescribed agents which inhibit statin metabolism by CYP450 enzyme, or affect activity of transporter proteins involved in statin cell flux, such as Organic anion-transporting polypeptides (OATPs) encoded by the SLCO1B1 gene [59].

Statins may be grouped due to differences in the enzymatic metabolism: lovastatin, simvastatin, and atorvastatin are mostly metabolized by the CYP3A4 isoenzyme [60], while fluvastatin, rosuvastatin, and pravastatin are primarily metabolized by the CYP2C9 enzyme, with a minor contribution from CYP2C8 (fluvastatin, pitavastatina), CYP2C19 (rosuvastatin), and CYP3A4 (fluvastatin) [60,61,62]. Most drugs are metabolized by CYP and approximately one half of these are metabolized by CYP3A4 [63], such as azole antifungals, macrolid antibiotics, protease inhibitors, tricyclic antidepressants, cyclosporine, amiodarone, and warfarin (Table 1), therefore, statins that are not metabolized by CYP3A4 have less risk of drug interactions [50].

## 5. Mechanisms

A number of pathophysiological hypotheses have been proposed to explain neuromuscular toxicity caused by statin therapy, nonetheless none of these have been unequivocally proved as underlying causes of SAMS.

### 5.1. Gene Regulation and Polymorphisms

Gene regulation studies conducted in the course of SAMS offer a glimpse of the pathways disturbed during this condition, although they do not distinguish the genuine effect of statins on muscle fibers from the adaptive response of the organism. The transcriptomic approach in patients with solid (i.e., proven by re-challenge) statin myopathy, compared to statin-tolerant patients shows that most of the differentially expressed genes act to balance some of the above-identified perturbed pathways. The most dysregulated, i.e., differentially expressed, genes were antisense ribonucleic acid (RNA) to the Homologous E6-AP Carboxy Terminus (HECT) domain E3 ubiquitin protein ligase 2 (HECTD2-AS1, a proinflammatory agent) and Uncoupling protein 3 (UCP3), a mitochondrial anion carrier protein with a postulated anti-oxidative stress activity. Other pathways involved (and upregulated) were Calmodulin (CALM), a calcium sensing protein that, when combined with calcium, inhibits RYR1 [64]. This mechanism has been postulated to balance the statin-dependent FK binding protein 12 (FKBP12) dissociation from RYR1 (described above) [57]. Among the downregulated genes, inositol 1,4,5-triphosphate receptor 2 (ITPR2) mediates calcium release from the sarcoplasmic reticulum [65], thus preventing mitochondrial senescence. In conclusion, transcriptomics shed light on the pathways activated to manage calcium signaling changes mediated by protein prenylation and Rat sarcoma virus-Guanosine triphosphatase (Ras-GTPase) activation.

Furthermore, several genetic polymorphisms, involved in statin metabolism and pharmacokinetics, may play a role in the pathogenesis of SAMS and explain individual variations in statin tolerance. Key genes are CYPs, uridine 5′-diphospho-glucuronosyltransferases (UGTs), SLCO1B1, and the efflux adenosine triphosphate-binding cassette (ABC) transporters, in particular ABCB1 and ABCG2. On this account, patients can be classified into extensive metabolizers, poor metabolizers, and ultra-rapid metabolizers [66,67,68]. Homozygous mutations associated with poor metabolism may lead, in theory, to higher plasma levels of statin and, consequently, to higher risk of adverse effects [48]. Gene polymorphisms in CYP enzymes may affect the severity of statin muscle toxicity and, thereby, symptoms, but the association remains uncertain [69,70]. Another possible mechanism is represented by ABC transporters (ABCB), which are involved in liver drug efflux. These proteins limit the access of statins in tissue compartments and promote hepatobiliary clearance [71]. It is possible that variations of the ABC genes could alter both pharmacokinetic and pharmacodynamic statins’ properties [72,73]. SLCO1B1, consistently associated with SAMS, encodes OATP1B1, which regulates hepatic uptake of statins. The Carnitine palmitoyl transferase (CPT) enzyme system plays an essential role in the transfer of long-chain fatty acids from the cytosol to the mitochondrial matrix [74]. CPT deficiency, an autosomal recessive disorder, is characterized by recurrent myalgia, rhabdomyolysis, and myoglobinuria after statin therapy and stress factors, such as heavy exercise, infection, and cold exposure [75,76].

### 5.2. Mitochondrial Disfunction

Some evidence correlates statin myopathy with mitochondrial damage [77,78,79,80]. CPT2, along with CPT1, deals with the oxidation of long-chain fatty acids in mitochondria. CPT2 deficiency results in autosomal recessive disease and it appears that these patients, or those carrying variants of this enzyme, develop SAMS more frequently [79]. In vitro analysis has shown that CPT2 mRNA can be disrupted by drugs, including statins, which cause rhabdomyolysis [81].

In vitro studies have shown a difference between lactone statins and acid statins, where the former appear to be more myotoxic than the latter, in particular simvastatin and fluvastatin [82]. Indeed, it appears that lactone sites can inhibit mitochondrial complex III and the respiratory capacity of the muscle cell [83]. On the other hand, other studies have shown that statin therapy does not affect mitochondrial membrane potential [83,84,85], and, therefore, statins are unlikely to act as mitochondrial decouplers. Therefore, further studies are needed to shed light on this mechanism.

Nine drugs have been shown to downregulate CPT2: promethazine, pindolol, loxapine, labetalol, haloperidol, clozapine, azacitidine, atropine, and amitriptylin. The contemporary use of statins and these medications may represent a confounding factor for the pathogenesis of myopathy.

### 5.3. HMG-CoA Reductase (HMGCR) Pathway

Mediated effects of statin inhibition on HMGCR perturb the mevalonate pathway. Whilst this perturbation has been linked to possible beneficial pleiotropic effects [86], importantly, statin-negative effects on muscle may be due to inhibition of biosynthetic pathways other than cholesterol synthesis. The impairment of these pathways could alter energy metabolism by reducing beta-oxidation of fatty acids and increasing intracellular lipids, determining the formation of lipid-filled vacuoles and fiber atrophy [87]. Mevalonate, which is a product of HMG-CoA reductase, is involved in the production of cholesterol, proteins, cofactors, and signaling molecules. Inhibition of HMG-CoA reductase induced by statin therapy results in lowering of several pathway intermediates, such as dolychols, prenylated proteins, electron transport chain proteins, and ubiquinone/coenzyme Q10, which is involved in oxidative phosphorylation [88].

All statins used in clinical practice decrease endogenous cholesterol synthesis by inhibiting HMG-CoA reductase enzyme and, therefore, all of them may potentially cause SAMS. However, statins diverge deeply in terms of effectiveness and chemical, physical, and pharmacokinetic characteristics.

Statins may be classified, according to their pharmacokinetic properties, in hydrophilic and lipophilic molecules, depending on their ability to dissolve in water or lipid-containing structures composed of a HMG-CoA analogue and a side chain structure that determines solubility. Lipophilic statins can easily enter cells by passive diffusion through membranes, resulting in a wide distribution in different tissues leading to more favorable cardiovascular outcomes. This statin’s class is mainly metabolized by CYP450 enzyme; conversely, hydrophilic ones remain associated with the polar membrane’s surface, needing protein carriers to penetrate cells with grater hepatoselectivity. A possible explanation of the increased risk of SAMS with lipophilic statins treatment may be due to non-selective diffusion of lipophilic statins into extrahepatic tissues, which supports greater Low Density Lipoproteins cholesterol (LDL-C) reduction and, on the other hand, major risk of SAMS for drug-drug interactions.

### 5.4. Protein Prenylation and Coenzyme Q10 (CoQ10)

The decreases in protein prenylation, CoQ10, and also cholesterol have been associated to SAMS. CoQ10 is a fat-soluble quinone [89] that plays an important role in mitochondrial energy metabolism and stabilization of muscle cell membranes [90,91] as a cofactor involved in the mitochondrial respiratory chain [92]. Several studies have showed that statins reduce CoQ10 plasma levels in skeletal muscle tissue of humans and rodents [79,93]. A deficit of CoQ10 does not seem to be associated with type of statin, intensity, or duration of treatment. Thus, statins may interfere with mitochondrial function, causing an impaired muscle performance, resulting in muscle damage. Structural abnormalities were detected in muscle biopsies of patients treated with statins, even when they did not experience any symptoms [10]. CoQ10 deficiency seems to affect directly electron transport chain, calcium signaling through the mitochondrial depolarization, and calcium release by the sarcoplasmic reticulum, resulting in caspase activation and induction of apoptosis [93,94,95,96,97]. Primary CoQ10 deficiency is an autosomal recessive condition associated with isolated myopathy, encephalopathy, nephrotic syndrome, cerebellar ataxia, and severe childhood multisystem disease [98]. A decrease in the concentration of CoQ10 in the circulation has been observed in patients treated with statins [99]; some studies have shown a moderate reduction in muscle CoQ10 [100], even if this association was not confirmed by other authors [101,102]. A recent RCT meta-analysis showed that CoQ10 supplementation does not reduce SAMS; a possible explanation is that the Q0 site of mitochondrial complex III is involved both in the transfer of electrons from CoQ10 to cytochrome C and in the off-target binding site for statin lactones [103]. Statins appear to reduce circulating CoQ10 and compete for its pharmacodynamic goal; therefore, the CoQ10 supplement alone can inadequately counteract the actions of statins. Larger studies are needed to confirm this conclusion [104].

A major pathway perturbed in statin therapy, and particularly in statin-induced myopathy, is post-translational protein prenylation [105]. Reduction of geranylgeranyl pyrophosphate generated by statin therapy reduces adenosine triphosphate (ATP) availability and reduces prenylation of small GTPases like Rab and Ras homology family member A (RhoA). RhoA-reduced prenylation led to mislocalization from cell membrane to the cytoplasm in fibroblast cells [106]. Statin-related apoptosis can be triggered by the reduction of Protein kinase B (PKB) phosphorylation [107], loss of Rab1 activity [108], and caspase-3 activation [106]. Of note, muscle damage induced by statin challenge can be prevented by geranyl supplementation [109].

Although intriguing, the statin-induced reduction of cholesterol in plasma membranes generating T-tubular system breakdown and sub-sarcolemmal rupture [110] has received limited attention, since the cholesterol pool in muscle is unchanged after statin therapy [111] and the cholesterol reduction obtained with Proprotein Convertase Subtilisin/kexin type 9 (PCSK9) inhibitors does not lead to similar adverse events [111,112].

### 5.5. Atrogin-1 Calcium Signaling and Glycine Amidinotransferase

Atrogin-1 is a muscle-specific ubiquitin protein E3 ligase playing a central role in inducing proteolysis and muscle atrophy. Atrogin-1 was found to be active in several conditions, such as cancer, kidney disease, and diabetes, but also during fasting [113]. A mechanism that is not well defined links atrogin-1 increased expression to statin therapy. Indeed, atrogin-1 expression was prevalent in muscle samples of statin-treated subjects [114]. In other studies, SAMS appear to be immune-mediated through the upregulation of Major Histocompatibility Complex-I (MHC-I) and other inflammatory mediators [115,116]. While atrogin-1 knockdown in zebrafish embryos prevents lovastatin-induced myotoxicity [117], clinical relevance of such a finding needs further investigation.

Mutations of some calcium trafficking genes, namely RYR1 and Calcium Voltage-Gated Channel Subunit Alpha1 S (CACNA1S), have been associated to severe conditions: anesthesia-induced malignant hyperthermia, central core disease [118], and multi-minicore disease [119] for the former and malignant hyperthermia and hypokalemic periodic paralysis for the latter. The prevalence of mutations within these two genes is increased in patients with statin-induced myopathy [120,121]. In ex vivo experiments, statin induced dissociation of the stabilizing protein FKBP12 from RYR1 in skeletal muscle, and this was associated with increased unnecessary calcium release sparks [122]. Even if these sparks induce pro-apoptotic signaling activation, statin therapy does not reduce muscle strength—this evidence questions the role of calcium trafficking and calls for other unidentified mechanisms to induce myotoxicity. Aside from this mechanism, the protective role of the minor allele of rs2819742 polymorphism in the RYR2 gene, the major Ca^2+^ channel protein in the membrane of the sarcoplasmic reticulum in cardiomyocytes, remains unexplained [123,124].

Creatine is a major source of energy in the muscle and Glycine amidinotransferase (GATM) is the enzyme catalyzing the first step of its synthesis. Different GATM genetic variants, rs9806699 and rs1719247, have been studied with genome-wide quantitative expression loci [125], by candidate gene analysis [126,127,128], and a meta-analysis [125,129,130], with inconsistent results. The evidence that, in the studies reporting a positive association, a minor allele is protective towards a rare condition like statin-induced myopathy, questions the reliability of such an approach.

### 5.6. Immunologically Mediated Statin Associated Myopathy

Immunomediated cell repair pathways might play a role in statin myopathy. A genetic variant in LILRB5 (leukocyte immunoglobulin-like receptor subfamily B) (rs12975366:T  >  C:Asp247Gly) has been reported to be associated with CK and Lactate dehydrogenase (LDH) levels. The mean CK levels of Asp247 homozygotes (T/T) were significantly higher, but the very same variant conferred an increased risk of generic myalgia, statin intolerance, and statin-related myopathy independently of the use of CK levels as diagnostic criteria [131]. On the pathophysiology of such an association, LILRB5 Asp247 is associated with Forkhead box P3 (FOXP3) expression in the spleen [132], which, in turn, is the master regulator of Treg (regulatory T cell) immune-suppressive activity.

In a minority of statin-intolerant patients, an autoimmune disease pattern can be postulated, since myopathy does not revert after drug discontinuation and, moreover, benefits from immunosuppressive agents [116,133,134]. Investigation on the mechanisms leading to such a condition identified, in a minority of patients, autoantibodies anti-HMGCR [135]. These patients suffered from a necrotizing myopathy with minimal lymphocyte infiltration, a distinct subtype of immune-mediated necrotizing myopathy [136]. Carriers of autoantibodies anti-HMGCR are often also carriers of Human leukocyte antigens (HLA)-DRB1*11:01 histocompatibility complex [137]. Even if strongly suggestive, the role of anti-HMGCR in pathogenesis of immune-mediated necrotizing myopathy has not been formally demonstrated, although statins upregulate muscle HMGCR expression in anti-HMGCR positive myopathy patients [135], anti-HMGCR induce muscle atrophy [64], and their plasma levels correlate with both circulating CK and stage of activity of the disease [138].

### 5.7. Neuromuscular Conditions

Statin therapy may exacerbate underlying neuromuscular disorders (including myasthenia gravis, dermal/polymyositis, inclusion body myositis, motor neuron disease, and MELAS (mitochondrial encephalopathy, lactic acidosis, and stroke-like episodes) [77,139,140,141]. Patients with hypercholesterolemia and untreated hypothyroid myopathy are also at increased risk for SAMS when on statin therapy. In these cases, however, SAMS resolves with discontinuation of the statin or initiation of hormone therapy [142,143,144]. Several metabolic myopathies have been associated with SAMS and very often patients are unaware of the disease until they start statin therapy [78]. Among the metabolic disorders associated with statin myopathy, those with an identified mutation include: Adenosine monophosphate deaminase (AMPD1) deficiency (formerly myoadenylate deaminase deficiency), Carnitine palmitoyltransferase 2 (CPT2) deficiency [79], accumulation of glycogen II [Pompe disease, Acid alfa glucosidase (GAA) deficiency] [135], V [McArdle disease, Muscle glycogen phosphorylase (PYGM) deficiency] [70] and IX [Muscle kinase b phosphorylase (PHKA1) deficiency] [145], malignant hyperthermia (RYR1, CACNA1S) [120,121], myoglobinuria recurrent infantile (LPIN1 mutation) [146], and myotonic dystrophy type I [Dystrophia myotonica protein kinase (DMPK) mutation] [69] and II [Cellular nucleic acid-binding protein (CNBP)] [147].

Cushing’s syndrome (CS) is characterized by a series of systemic complications, including hypertension, hyperlipidemia and glucose intolerance, and hypercoagulability, all associated with increased cardiovascular risk [148]. Lipid abnormalities, including elevated total cholesterol, LDL-C, and triglycerides, are reported in 40–70% of patients with CS and may persist after surgical remission. Dyslipidemia, secondary to chronic glucocorticoid excess, represents a critical contributor to increased cardiovascular complications, such as vascular atherosclerosis, coronary artery disease, and heart failure [149]. Treatment of persistent hyperlipidemia should be conducted according to the lipid-lowering treatment goals of 2019 ESC/EAS guidelines. Furthermore, patients requiring medical treatment for persistent hypercortisolism present specific challenges, according to the selected therapeutic agent. For example, treatment with the adrenolytic drug o,p’DDD (mitotane) is associated with a significant increase in cholesterol levels and the use of ketoconazole, a potent inhibitor of cytochrome P450 3A4 (CYP3A4), may significantly increase plasma concentrations of lipophilic statins simvastatin and atorvastatin), increasing the risk of complications. In summary, hyperlipidemia should be aggressively treated in patients with CS in view of the increased cardiovascular morbidity and mortality associated with this disorder. Ketoconazole, an antifungal agent, is the most widely used drug in the pharmacotherapy of CS. It is an adrenal steroidogenesis inhibitor, acting on several steroidogenesis enzymes, including the cholesterol side-chain cleavage complex, 17,20-lyase, 11β-hydroxylase, and 17β-hydroxylase [150]. Ketoconazole is also an inhibitor of cholesterol biosynthesis, acting directly by blocking the conversion of methyl sterols to cholesterol and indirectly by suppressing cholesterol synthesis, reducing total, intermediate-density, and LDL-C by approximately 25% [151,152,153].

### 5.8. Pain Threshold

Pain threshold can be a determinant for statin-related myopathy when myalgia is a diagnostic criterion. Genetic variants of serotonin receptors 3B and 7 (namely rs2276307 and rs1935349) are significantly related to myalgia score, but not to serum CK levels [154]. Consistently, when CK was a parameter to diagnose statin-related myopathy in Genome-Wide Association Study (GWAS), these findings were not replicated [44,123,129]. 

### 5.9. Physical Exercise

Some authors have reported that exercise together with statins can exacerbate or even trigger SAMS [155,156]. After a training session, CK levels appear to increase more in statin-treated athletes than in athletes not on therapy [157]. Furthermore, the association between high CK levels and age was found only in athletes on statin therapy [157]. However, a systematic review of the literature reports conflicting results on the ability of statins to impair performance during exercise [158]. The increase in circulating levels of specific microRNAs (myomiRs) has recently been indicated as a potential biomarker of muscle quality [159]. It has been described that an increase in the levels of some myomiRs is related to intense exertion (miR-1, miR-133a, miR-206), and one in particular (miR-499-5p) seems to increase only in athletes on therapy with statins [160]. These observations suggest a role for epigenetics in muscle damage and confirm a synergy between statins and exercise by identifying a potential biomarker for identifying patients with exercise-exacerbated SAMS. Interestingly, some animal studies have suggested that gradual training may improve muscle tolerance to statin exposure [122,161]. Therefore, further work is needed in this area to clarify this topic.

### 5.10. Vitamin D

A correlation between vitamin D deficiency and SAMS has been suggested, but the impact of statin on vitamin D level is still controversial [162]. The vitamin D family is a group of fat-soluble secosteroids that regulates calcium and phosphate concentrations and bone mineralization, with cholecalciferol (vitamin D3) and ergocalciferol (vitamin D2) being the main forms in humans. Vitamin D is primarily obtained through the conversion of 7-dehydrocholesterol (synthesized endogenously from cholesterol) to cholecalciferol by Ultraviolet B (UVB) light, although cholecalciferol and ergocalciferol can also be acquired from food [163].

Vitamin D undergoes sequential hydroxylation, first to 25-hydroxycholecalciferol/25-hydroxyergocalciferol, which are the major circulating (but also inactive) forms, and then to 1,25-dihydroxycholecalciferol (calcitriol)/1,25-dihydroxyergocalciferol [collectively 1,25 (OH) 2D], which are the biologically active vitamin D forms [163]. 1,25 (OH) 2D binds to a vitamin D receptor, found in multiple tissues, including bones, kidneys, intestines, parathyroid glands, and skeletal muscle, and through it mediates genomic and non-genomic effects [163,164]. The role of statins on vitamin D levels is controversial [165]—considering the inductive effect of 1,25 (OH) 2D on CYP3A4, vitamin D supplementation reduces atorvastatin [166]; however, low vitamin D levels can attenuate the lipid-lowering response in atorvastatin-treated patients, probably due to the inhibitory effect of vitamin D derivatives on HMGCR [163]. Vitamin D deficiency causes osteomalacia/rickets, muscle weakness, and myopathy. A meta-analysis has confirmed that plasma vitamin D levels are significantly lower in statin-treated patients with myalgia, compared to those not affected by SAMS [167]. Furthermore, several (non-randomized) clinical studies have reported that vitamin D supplementation reduces statin-related myotoxicity incidence in patients who had previously reported statin intolerance that were undergoing rechallenge therapy [168,169,170,171,172].

## 6. Mechanisms of Non-Statin Therapies for Hyperlipidemic Patients

SAMS management includes different options, ranging from lifestyle modifications, rechallenge therapy, and alternative medications use. A healthy lifestyle, including exercise and foods rich in mono-polyunsaturated fats and plant sterols, can lower LDL-C by 10–15% [173,174]. The cornerstone in SAMS treatment is the rechallenge therapy with a lower dose of the same statin or an alternative statin. Another option could be the “non-daily dosing strategy”, which is associated with lower drug levels and larger swings in drug concentration. This strategy works better with atorvastatin and rosuvastatin, which have longer elimination half-lives and can be administrated every other day or even less frequently [25,175].

If these strategies are not tolerated and LDL-C persists above target, other therapeutic options should be considered. Ezetimibe reduces absorption of dietary and biliary cholesterol in the proximal small bowel. It can reduce LDL-C by 15–20% with few side effects [176] and has an impact on cardiovascular event reduction [177]. The Improved Reduction of Outcomes: Vytorin Efficacy International Trial (IMPROVE-IT) assessed that adding ezetimibe to low-dose simvastatin in post-acute coronary syndrome patients provided a greater prevention of cardiovascular events than simvastatin alone [178].

Among the most commonly used non-statin medications, there are monoclonal antibody PCSK9 inhibitors that inactivate the liver PCSK9. PCSK9 regulates ligand binding and degradation of the hepatic Low Density Lipoproteins receptor (LDL-r) and its inhibition can lower circulating LDL-C by 50–70% with or without concomitant statin therapy [179,180]. In the Fourier trial patients who did not achieve treatment goals with the maximum tolerated dose of statin and ezetimibe, adding evolocumab reduced cardiovascular events by 11.3% [181]. In the Odyssey trial, involving patients who experienced an acute coronary syndrome 1 to 12 months earlier, SAMS occurred less frequently with alirocumab (32,5%) than atorvastatin (46%) [181]. Bile acid sequestrants (resins) can reduce LDL-C levels by 15–25% and can reach a 30–35% reduction in combination with ezetimibe [111,182]. Despite having a stronger action on triglycerides, fibrates lower LDL-C levels as well, and play a role in the reduction of cardiovascular risk in dyslipidemic patients [183,184]. A number of complementary therapies, such as CoQ10 and vitamin D supplementation, have been suggested to improve statin tolerability but data do not strongly support the COQ10 supplementation and the evidence for the effectiveness of vitamin D is still controversial [185,186,187,188,189,190].

Bempedoic acid, a once daily oral drug that inhibits ATP citrate lyase, a component of cholesterol biosynthesis pathway, promises to bring a new perspective in the treatment of dyslipidemia. Bempedoic acid is a prodrug activated by acyl-CoA (Coenzyme A) synthetase-1, an enzyme located in hepatocytes but not in skeletal muscles; this may prevent SAMS [191]. The Cholesterol Lowering via Bempedoic Acid, an adenosine triphosphate-citrate lyase (ACL)-Inhibiting Regimen (CLEAR) Serenity study, a double-blind, placebo-controlled trial, enrolled 345 patients with hypercholesterolemia and a history of intolerance to at least two statins with one at the lowest available dose, both in primary and secondary prevention. The bempedoic acid treatment arm demonstrated a 23.6% reduction in LDL-C levels versus a 1.3% reduction in the placebo treatment arm (*p* < 0.001). The findings of CLEAR Serenity demonstrated that bempedoic acid could be considered a safe and effective LDL-C-lowering therapy for patients with a history of statin intolerance [192]. In fact, during the study, myalgia occurred in 4.7% and 7.2% patients receiving bempedoic acid or placebo, respectively. Bempedoic acid could offer a safe and effective oral therapeutic option for lipid lowering in patients who cannot tolerate statins.

Recently, new therapeutic approaches based on physiological mechanisms of gene silencing, a post-transcriptional process that regulates cells gene expression by turning off a selected gene, have raised increasing interest in many fields. In particular, both Antisense oligonucleotides (ASO) and small interfering RNA (siRNA) technologies have been developed in order to control the expression of specific genes playing key roles in lipid metabolism [193]. A promising innovation is represented by inclisiran, a long-acting synthetic siRNA directed against PCSK9 that has been shown to significantly decrease hepatic production of PCSK9 and cause a marked reduction in LDL-C levels [194].

Lifestyle plays a known key role in both primary and secondary prevention, affecting different cardiovascular risk factors, including cholesterol level. An adequate non-pharmacological action, such as nutrition and physical activity (30 min a day of rapid walking 4 to 6 Km/h) [195], should be tried for at least three to six months in intermediate-risk patients before statin treatment and may be an additional tool in LDL-C lowering in statin-intolerant patients. Recent guidelines [196,197] recommend a number of possible interventions: (1) a diet poor in trans-saturated fatty acids and saturated fatty acids, including full fat content milk and dairy products, red meat, and fatty cold cuts; (2) a diet poor in simple sugars and refined cereals; (3) increased consumption of fruits, vegetables, legumes, whole grains, and olive oil; (4) increased consumption of blue fish, nuts, seeds, and linseed oil, which are rich in omega-3. It should be noted that, while fresh fruit consumption is recommended, grapefruit and its juice are usually avoided while on lipid-lowering therapy with statins, because this fruit is rich in furanocoumarins, which inhibit the activity of an isoform of the cytochrome P450 superfamily (CYP3A4) and, therefore, increases the availability of some statins (atorvastatin, simvastatin, lovastatin). This effect was found to be related to the amount and timing (in relation to drug assumption) of grapefruit. A recent study showed a minimal risk of developing side effect in patients treated with statins that were given 250 mL of grapefruit juice per day, while the lipid-lowering effect of statins was increased, LDL-C levels were reduced by 37% with statins alone and by 48% with the combination of statin and grapefruit juice; this synergistic action also carried a further 10% cardiovascular risk reduction as compared to drug therapy alone [198].

In addition, a number of food components and functional foods have proven or possible lipid-lowering properties. These include dietary fiber, omega-3 polyunsaturated fatty acids (n-3 PUFA), phytosterols and phytostanols, soy proteins, fermented red rice, berberine, and polycosanols. The Reduction of Cardiovascular Events With Icosapent Ethyl-Intervention Trial (REDUCE-IT), a recent trial published by the New England Journal of Medicine, highlighted for the first time a significative reduction of risk of major ischemic events achieved with a food component among hypertriglyceridemic patients treated with 2 g (n-3) icosapent ethyl twice daily [199].

## 7. Conclusions

Statins are generally safe and highly effective pharmacological tools that reduce the burden of atherogenic serum lipoproteins and cardiovascular risk. SAMS are the most common side effect associated with low compliance and discontinuation of lipid-lowering treatment. A step-by-step approach, including careful examination of all other possible factors that may increase risk of statin intolerance, might help patients’ compliance to lipid-lowering therapy. SAMS management includes different options, ranging from lifestyle modifications, tested for at least three to six months; rechallenge therapy or switching to a different statin; and alternative medications, such as ion-exchange resin, nutraceuticals, ezetimibe, and PCSK9 inhibitors. A promising perspective is offered by recently developed drugs, such as bempedoic acid and inclisiran. Large outcome trials are needed to develop a consensus on a treatment plan that weighs the costs and benefits of statin use versus other pharmacological and non-pharmacological options for long-term reduction of atherosclerotic cardiovascular disease events.

## Figures and Tables

**Table 1:** Drugs potentially interacting with statins to increase myopathy risk.

CYP3A4 Inhibitors	CYP2C9 Inhibitors	OATP1B1 Inhibitors	Glucuronidation Inhibitors
Protease inhibitors Macrolide antibiotics Azole antifungals Non-dihydropyridine calcium channel blockers (diltiazem and verapamil) Antidepressants (nefazodone) Cyclosporine Amiodarone Drinking large amounts of grapefruit juice or cranberry juice (N250 mL per day)	Fluconazole Amiodarone Fenofibrate (mild-to-moderate effect)	Cyclosporine Protease inhibitors	Gemfibrozil

## Data Availability

Not applicable.
